# What do you think it means? Using cognitive interviewing to improve measurement in implementation science: description and case example

**DOI:** 10.1186/s43058-024-00549-0

**Published:** 2024-02-14

**Authors:** Zabin Patel-Syed, Sara Becker, Miranda Olson, Hailey Rinella, Kelli Scott

**Affiliations:** Northwestern University Feinberg School of Medicine, Institute for Public Health and Medicine, Center for Dissemination and Implementation Science, Chicago, USA

**Keywords:** Implementation, Pragmatic measures, Assessment, Cognitive interview, Case report

## Abstract

Pragmatic measures are essential to evaluate the implementation of evidence-based interventions. Cognitive interviewing, a qualitative method that collects partner feedback throughout measure development, is particularly useful for developing pragmatic implementation measures. Measure developers can use cognitive interviewing to increase a measure’s fit within a particular implementation context. However, cognitive interviewing is underused in implementation research, where most measures remain “homegrown” and used for single studies. We provide a rationale for using cognitive interviewing in implementation science studies and illustrate its use through a case example employing cognitive interviewing to inform development of a measurement-based care protocol for implementation in opioid treatment programs. Applications of cognitive interviewing, including developing a common language with partners and collecting multi-level feedback on assessment procedures, to improve measurement in implementation science are discussed.

**Supplementary Information:**

The online version contains supplementary material available at 10.1186/s43058-024-00549-0.

Contributions to the literature

- Measurement concerns in implementation science are among the most significant barriers to advancing the field.
- Previous studies suggest that implementation measures are often used in single studies, high burden, and developed without partner input.
- There remains limited guidance on methods to develop pragmatic measures.
- To address this gap, we provide a brief overview of cognitive interviewing, a qualitative method that uses partner feedback throughout the measure development process.

## Background

Measurement issues in implementation science are among the most critical barriers to advancing the field [7, 9, 21–23, 30]. Measures developed and tested in efficacy trials may not be feasible in service systems, and the widespread use of “homegrown” implementation measures limits generalizability of study findings [12, 25]. Implementation science is especially vulnerable to measurement issues given the rapid growth of the field and the need for multi-level measurement in diverse health contexts (e.g., community mental health treatment, medicine, etc.) [31].

Measure development involves conceptualization (identifying measurement gaps and relevant constructs for a target population); development (generating measure content and administration procedures); and testing (assessing psychometric properties) [5]. Psychometric testing has received the most attention in the implementation science literature [20, 26]. However, implementation partners—treatment developers, implementation researchers, community leaders—are unlikely to select measures based on psychometric evidence alone [13, 14, 29]. Emphasis must also be placed on a measure’s pragmatic qualities, goals for use, and translatability to clinical practice [34].

Glasgow and colleagues [13] recommended guidelines for pragmatic implementation measures. Based on a review of the literature, the authors noted that pragmatic measures have four key characteristics: importance to partners; low burden for respondents; actionable; and sensitivity to change. Extending this work, Stanick and colleagues [34] interviewed implementation science experts and identified the following three characteristics as priorities: integration with an electronic/health record, facilitation of guided action (e.g., selection of an intervention), and low-cost. This work contributed to the development of the Psychometric *and* Pragmatic Evidence Rating Scale (PAPERS) for evaluating implementation measures [21, 22]. However, there remains limited guidance on methods for developing pragmatic implementation measures to be used across different contexts.

Implementation measures must balance both psychometric and pragmatic quality. To attain this balance, we advocate that implementation scientists routinely use cognitive interviewing, a qualitative method that collects partner feedback throughout measure development [40]. Cognitive interviewing is uniquely suited to address measurement concerns in implementation science for four key reasons. First, implementation measures often evaluate efforts that engage diverse partners across multiple levels (patient, provider, organization) [1, 35]. Cognitive interviewing can reveal whether measure content is relevant across partner groups and inform tailoring as needed. Second, cognitive interviews can help assess psychometric and pragmatic characteristics, including a measure’s construct validity, training burden, relevance, and usefulness across different contexts. Third, unique to implementation research, in which context is paramount [4, 11, 28], cognitive interviews can be used to collect partner feedback on measure administration procedures. Cognitive interviews can assess partner preferences for a measure delivery platform (e.g., electronic or paper), measure format (e.g., time, length, multiple choice versus free response), and strategies to integrate the measure with a clinical setting’s workflow (e.g., when, and how often to administer a measure), all of which can enhance a measure’s utility and scalability. Finally, collaborative research techniques like cognitive interviewing can be used to center partner perspectives, which can promote equitable partnership-building and increase buy-in [36].

To advance the development of psychometrically and pragmatically valid tools, we advocate for the widespread use of cognitive interviewing in implementation science studies. We first provide a detailed overview of cognitive interviewing theory and the stages of cognitive interviewing. We then provide a case example from an ongoing implementation trial to demonstrate how cognitive interviewing can be used to develop a pragmatic measure and to design a measure administration protocol [32]. We conclude with reflections on how cognitive interviewing can be used to improve measurement in implementation science.

## Cognitive interviewing: overview of theory and techniques for use in implementation science

During a cognitive interview, implementation partners verbalize their thoughts as they evaluate measure questions and provide responses [2, 40]. As the partner reads a measure aloud, an interviewer uses intermittent verbal probes to elicit their response process (concurrent interviewing) or has the partner verbalize their thoughts after completion (retrospective interviewing). Interviews may be used to identify constructs that partners value and consider important to assess (concept elicitation) or to revise an existing measure (debriefing). This method is used widely in other areas such as survey methodology and health outcome measurement (e.g., patient-reported outcomes in clinical trials), and by organizations like the United States Census Bureau [6, 16, 27] for measure development.

Cognitive interviews can be tailored to the goals of an implementation study. Given implementation research often includes a broad range of academic and community partners, interviews can be tailored for specific partner groups, to assess specific parts of a measure (e.g., instructions, terms, response options), to examine the relevance of the measure, or to evaluate administration procedures. In addition to its flexibility, cognitive interviewing can produce informative data even with small sample sizes (e.g., 5–10 interviews and a 15–30-min interview period) [40], which is particularly useful for resource-constrained implementation efforts.

### Cognitive interviewing theory

Drawing on cognitive psychology, cognitive interviewing frameworks propose that a partner follows a four-stage mental model: (1) comprehension; (2) memory retrieval; (3) judgement; and (4) response [10, 17, 37]. At the comprehension stage, the goal is for the partner to interpret measure content (e.g., instructions, items, response options) as intended by the developer [39]. Misunderstandings may result from confusing or complex wording, missing information, inattention, and unfamiliarity with terminology. Measurement error due to comprehension issues [40] is especially likely in implementation science where it is well documented that users are often unfamiliar with key constructs [3, 8]. For example, the question, “Recently, how many days have you participated in a training on evidence-based practice?” presumes the partner comprehends key terms about time reference (“recently”), implementation strategy (“training”), and a construct (“evidence-based practice”). If the partner is unfamiliar with these terms, they may not understand what types of training activities and intervention to include when responding to the question, which contributes to measurement error.

Next, to recall an answer, the partner must draw on information in memory. Several factors influence the memory retrieval process including a partner’s past experiences and the number and quality of memory cues provided, such as the time anchor (e.g., “recently”) and examples (e.g., participation in a workshop versus ongoing training) [10]. Third, the partner must integrate the information presented and form a judgement [40]. Previous studies indicate that decreasing item complexity (e.g., length, vocabulary) may facilitate decision-making, leading to more accurate self-reports [18]. In the example provided, researchers could consider changing the time anchor, replacing the general term “evidence-based practice” with a specific intervention, and simplifying the question (“Over the past month, did you attend a workshop on cognitive behavioral therapy?”).

In the final stage, the partner selects an answer and communicates it to the interviewer [17, 40]. It is important to consider how response options are provided, specifically the type of scale used (e.g., Likert scale, rank order, multiple choice, open-ended), the direction of response options (e.g., “Strongly Disagree to Strongly Agree” versus “Strongly Agree to Strongly Disagree”), and whether the partner can meaningfully differentiate among the response options. In sum, cognitive processes involved in recall and recognition are affected by how measure content is presented, and these factors warrant consideration in measure development.

### Cognitive interviewing techniques

Several cognitive interviewing techniques, generally categorized as think aloud and verbal probing [10, 40], may be used. In think aloud, the interviewer takes an observer role and asks a partner to spontaneously verbalize their thoughts as they respond to questions. In verbal probing, the interviewer takes a more active role by asking a partner pointed follow-up questions after each response. Probes may be general (Does this question makes sense?) or item-specific (What do you think the term “evidence-based practice” means?). Probe selection can be standardized/pre-planned or applied flexibly in response to the partner (You hesitated to answer, can you tell me why?). The goals of the implementation study will guide probe selection. Table 1 presents key goals of cognitive interviewing and probes to elicit implementation relevant feedback.

**Table 1:** 4-stage cognitive interviewing model and example verbal probes for implementation studies

Cognitive Interviewing Model	Example verbal probes
***Comprehension*** Clarify understanding of constructs, define terms, paraphrase questions	-Does that make sense? -What does “evidence-based practice” mean to you? -What do you think is the difference between “sustainability” and “maintenance”? -How would you ask this question in your own words? -Are there other words a provider may use to describe “evidence-based practice”? -How would you explain “organizational climate” to a clinician?
***Memory Retrieval*** Examine the utility of memory cues provided (e.g., time anchor, examples)	-Do you need more response options to help you answer the question? -Is the “6-month” timeframe used in this question useful? -Would having more examples of specific “evidence-based practices” in the instructions be helpful?
***Judgement*** Assess confidence judgements	-Are there other questions you would include on a clinician knowledge test about evidence-based practice? -Do you think other clinicians would answer this question in the same way? -How sure are you that you attended 5 days of training on evidence-based practice?
***Response*** Elaborate upon the response process	-Why did you choose “Agree” instead of “Strongly Agree”? -What types of trainings were you thinking about when you said you went to “2 days of training on evidence-based practice”? -Are you including time you spend in clinical supervision in your answer about evidence-based practice training?
***Implementation Research*** Collect partner feedback on administration procedures	-How often do you think your [staff/clinicians/patients] should complete this measure? -Do you think it would be better for [staff/clinicians/patients] to complete this measure in private or in a group setting? -Are there other supports (e.g., internet access, survey platform, additional training) your site needs to complete this measure?

Cognitive interviewing experts recommend using a structured or semi-structured protocol to guide data collection (see [40]). The protocol typically includes study-specific interview techniques (e.g., standardized probes) and administration information (e.g., use of technical equipment). For implementation studies, the cognitive interview protocol may also include several key additions: (1) probes to elicit multi-level partner perspectives (e.g., asking a clinical provider: What factors may affect how a patient would answer this question?,asking a clinical supervisor: Do you think clinicians would need additional training to administer this question?); (2) definitions of terms to facilitate shared understanding between partners (e.g., Can you describe what evidence-based practice means in your own words?); and (3) instructions on how to tailor probes for specific partner groups (e.g., clinic supervisors versus front-line providers). Given the multi-level nature of implementation studies, analyzing data at the item- *and* partner-level may reveal important patterns in terms of conceptual themes, informant discrepancies, targeted revision areas, and measurement feasibility barriers. These patterns can inform subsequent refinements to the measure and measure administration protocol to enhance the usability and scalability in real-world contexts.

## Cognitive interviewing case example in ongoing implementation science project

Our team is currently employing cognitive interviewing to develop a pragmatic measurement-based care (MBC) tool. MBC is an evidence-based practice that involves the systematic administration, review, and discussion of patient assessment data to inform treatment decisions [19, 33]. Few measures to assess patient progress in opioid use disorder treatment exist [24]. To address this need, the Director of the National Institute on Drug Abuse (NIDA) put forth a call to develop pragmatic measures of opioid use disorder symptoms and overdose risk. In response to this call, the NIDA-funded Measurement-Based Care to Opioid Treatment Programs (MBC2OTP) Project (K23DA050729) aims to develop a pragmatic overdose risk measure and measure administration protocol [32]. A preliminary 22-item measure was drafted by members of our study team based on published recommendations from the NIDA Director and colleagues and the DSM-5 diagnostic criteria for opioid use disorder [24]. Cognitive interviews are being used to collect partner feedback on measure content (symptoms, impairment, frequency of opioid use), format (open-ended questions versus multiple choice, preferred length, scoring), and administration procedures to inform implementation in community opioid treatment programs (OTP).

Multi-level partners are being recruited via email for cognitive interviews in two rounds. In the first round, relevant partners include program leaders who would decide whether to introduce the measure at an opioid treatment program, clinical supervisors who would oversee the training and supervision of counselors in measure administration, and front-line counselors who would deliver the measure to a patient. The second round of interviews focus on patients who would complete the measure in treatment. Eligibility requirements include English fluency and staff employment at the opioid treatment program for at least 3 months. No other exclusion criteria are used. Exclusion criteria are purposefully minimal to capture a range of diverse partner perspectives.

During the interview, three female researchers trained in cognitive interviewing present partners with the measure draft and ask them to answer each question aloud. We then apply the four-stage cognitive model to assess participant comprehension, memory retrieval, judgement, and response. First, in the comprehension phase, we assess whether partners comprehend the question and all the embedded constructs. For instance, our draft tool contains the item, “What typical dose of opioids do you take?” Ensuring comprehension requires us to assess whether a patient understands what opioids are and if they are aware of their average levels of opioid use.

Next, we assess the partner’s ability to recall an answer by drawing on information in memory. For example, we assess whether a patient’s response to the question about typical opioid use may differ based on whether they are experiencing withdrawal symptoms and if they would value examples of opioids in the item wording.

Third, we ask the partner to think aloud and describe how they are answering the question, so that we can assess how they form a judgment [40]. We also assess whether item complexity (e.g., length, vocabulary) seems appropriate or whether the item can be simplified to facilitate more accurate self-reports [18]. In the example provided, we ask whether participants might prefer a different time anchor or simpler wording of the question (“Over the past month, did you use more opioids than usual?”).

In the final stage, we ask the partner to communicate their final response to the question to the interviewer [17, 40]. In our cognitive interviews, after a partner provides a response to one of the MBC items, we elicit their feedback on how the question is presented using verbal probes, which are outlined in a semi-structured protocol [10, 40]. We use both general probes (Does this question makes sense?) and item-specific probes (What do you think the term “dose” means?) that are applied flexibly in response to the partner (You hesitated to answer, can you tell me why?). Importantly, our cognitive interview protocol uses supplemental open-ended questions to collect feedback on the ideal measure administration procedures to facilitate implementation of the protocol into the organizational workflow. Specifically, we elicit feedback on assessment frequency (how often the measure should be administered), administration context (group vs. individual counseling; in-person vs. telehealth sessions), and preferred administration method (electronic health record vs. tablet vs. pen and paper). Additionally, as an extension of typical cognitive interviewing, partners are asked to reflect on the types of implementation supports likely needed. Table 2 presents the four steps of cognitive interviewing currently being applied in the MBC2OTP study. Additional file 1 presents the full cognitive interview script used in the MBC2OTP study.

**Table 2:** Cognitive interviewing applied to the development of a pragmatic measure and administration protocol: The MBC2OTP case example (K23DA050729)

**Cognitive Interviewing Steps**	**MBC2OTP example**
1. Introduce partners to the purpose of the cognitive interview	1. Partners are shown the MBC2OTP pragmatic measure via videoconference screen share 2. An interviewer reads a welcome script which includes an overview of interview goals, a definition of the measurement goals, technical procedures (e.g., audio-recording, transcription, etc.)
2. Ask partners a question and record the response	1. Partners are asked to administer the measure as they would in a counseling session with a patient 2. Partners are asked to consider how the assessment data collected could inform their treatment approach
3. Use a series of verbal probes corresponding to the 4-stage cognitive interviewing model: (1) Comprehension (2) Memory Retrieval (3) Judgement (4) Response	Scripted probes are used to assess question clarity, relevancy, and alignment with program goals Interviewers note if partners need any part of the question repeated, if partners have difficulty using any of the response options provided or ask for clarification prior to providing their response. Partners are also provided the opportunity to give general feedback about each question including why they answered the way they did, how easy it was to provide an answer, and whether the language/content was appropriate
Repeat steps 2–3 for each item	
4. Collect partner feedback on measure administration protocol	Open-ended questions are used to examine measure administration procedures: 1. [Question content] How well do you feel the questions captured the important things to ask to assess treatment progress? 2. [Measure format] What are your thoughts on measure length? How long would you prefer that the measure take? 3. [Measure administration] How frequently should patients complete a measure like this? 4. [Measure fit for group counseling] Should this measure be completed prior to or during a group counseling session? 5. [Current treatment workflow] How easy would it be to make a measurement-based care tool available for use in your electronic health record?

One-on-one partner interviews are currently being conducted via videoconference, are audio-recorded, and transcribed. Transcripts are being analyzed by three independent coders (ZPS, HR, and KS) to thematically identify areas for revision using NVivo. Using a reflexive team analysis approach [15], the study team meets weekly to establish consensus and resolve coding discrepancies. Reflexivity in qualitative analysis refers to the process by which the researcher identifies and reflects on the impact they may have (i.e., their own assumptions and biases) on the data being collected and analyzed in a study. The reflexive team analysis approach was selected to enable the coding team to iteratively reflect on their roles as researchers who are unfamiliar with the OTP context, as well as how this outside role may have impacted data collection, analysis, and interpretation.

Suggested revisions are being analyzed by item and partner background. Cognitive interviews will be continued until a representative sample is obtained from each participating OTP, defined as interview completion with all eligible partners who consent at each site. Data from these initial interviews will inform iterative development of the pragmatic MBC measure and measure administration protocol. Discrepancies and conflicting views across different partner groups (e.g., leaders and patients) will be resolved via collaborative co-design meetings with representatives from each OTP and the research team following interview completion. Results from the qualitative data analysis will be presented to OTP representatives, and consensus discussions will be held to make final decisions about conflicting feedback on each measure item.

To date, we have conducted 13 first-round 30 to 60-min cognitive interviews with participants from three opioid treatment programs (*n* = 6 opioid program leaders; *n* = 3 clinical supervisors; *n* = 4 front-line counselors). Data collection is ongoing and an additional five opioid treatment programs will be recruited to participate in the MBC2OTP study. Table 3 presents illustrative data gathered from the multi-level partners thus far to highlight how cognitive interviewing can be used to elucidate feedback on potential measure refinements as well as workflow administration.

**Table 3:** Illustrative multi-level partner feedback to inform revisions to a pragmatic measure and measure administration protocol for community opioid treatment programs

**Pragmatic measure**					
**Question Tested**	**Opioid Treatment Program Leader (001)**	**Clinical Supervisor (002)**	**Front-line Counselor (003)**	**Strategic Decision Made**	**Preliminary Item Revision**
**1. Have you use opioids, sedatives, or cocaine in the past 3 months?** a. Yes b. No **1a. If yes, how often have you used them?** a. 5–6 times per week b. 3–4 times per week c. 2 times per week d. 1 time per week e. 1–3 times a month f. Less often	“I wonder about the word sedatives. I think people, our population, might need a greater explanation of what counts as a sedative. So like, do we mean benzos, tranquilizers, barbiturates, alcohol? You know, how broad are we going?”	“I’m not sure really what we’re trying to capture, if we’re trying to capture use in the last 12 months, 3, or 6. To me, if you’re trying to capture if somebody needs detox or if somebody needs a certain level of care, the 3, 6, and one year interval because 6 months in the addiction world, if you have not used then you’re in remission.”	“It is a relevant question and it’s an opioid treatment program, so obviously that’s the first question you want to ask. I ask that even during my sessions, so my sessions group, doing an intake, with any interaction, the first question I ask is ‘have you used in the past 30 days?”	-Focused question to only opioid use -Simplified response options to daily, weekly, or monthly use -Added skip logic on survey to display frequency question only if respondent selects “yes” to use	**Have you used opioids in the past 3 months?** a. Yes b. No **If yes, how often have you used opioids?** a. Daily b. Weekly c. Monthly
**2. Have you ever experienced problems as a result of the time you have spent to get opioids?** a. Yes b. No	“That sounds a little confusing to me. I know what they mean, but the problems we’re trying to assess are specifically a result of the time spent trying to track down the substance. I just wonder about the wording of it.”	“Personally to me, my clients’ comprehension is way lower than that with that question, so rewording it and it basically: did you get in trouble.”	“That’s okay. Not bad.”	- Simplify question wording - Reconcile discrepant partner feedback (e.g., confusing question versus no revisions needed)	**Have you gotten in trouble because of your opioid use?**
**Measure administration protocol**					
**Measure Administration Question**	**Opioid Treatment Program Leader (001)**	**Clinical Supervisor (002)**	**Front-line Counselor (003)**	**Strategic Decision Made**	
**What are your thoughts about the measure/question content?** • Any questions that should be added? Anything that should be deleted?	“I feel like the old sheets that we had them do in that old opioid cessation group were like ‘Did you do anything this week to try to use less or try to avoid using opioids?’ ‘What was it?’ ‘Did it work?’”	“So yeah, you really got to figure out what you’re trying to say in the first question [question 1] for the second one [question 1a] to make sense, but I would get rid of [response option] F [less often].”	“Maybe one question that I might want to add is ‘are you using both? Are you using opiates and cocaine at the same time?”	- Review existing measures at treatment programs to identify overlapping content and reduce measure length - Combine response options for measure simplification - Add questions about polysubstance use - Update measure administration protocol with a section on training - Pilot test revisions in additional cognitive interviews	
**What are your thoughts about measure administration procedures?** • For example, how frequently should patients complete a measure like this?	“It depends a bit on the periodicity, I think. I’m not sure that all of these questions need to be administered weekly, for example. Or maybe having it set up in such a way that easily flows like ‘If no, skip.’	“I don’t see it as a pre and post that all… I don’t really see it in any other way other than an intake.”	“I would like to administer something like this during their quarterly assessments. So, every three months we do an assessment.”	- Add skip logic so only relevant items are administered - Revise measure administration to occur at each completed counseling session, at program intake, and during required quarterly assessments - Pilot test revised measure administration plan in additional cognitive interviews	
**How do you feel a measure like this would fit into a typical group counseling session at your treatment program?**	“The other thing I wonder about, in a group setting I wonder if patients would feel uncomfortable disclosing the answers…And I just wonder, in a group setting, how would we navigate all that data entry?”	“In a group? No, absolutely not. This is very individualistic, very personal. This is very shameful, this is– no. Absolutely not.”	“So, their answers will help you know what topics to treat in-group. As a group facilitator, because not all patients in your group are your patients, right? If you give this assessment in group to get the response, you are able to communicate with their individual clinicians. Maybe there is something that they’re telling their individual clinicians that you’re not aware of as a facilitator.”	- Revise measure administration plan to administer to patients *prior* to their counseling sessions - Revise measurement-based care protocol for use in individual instead of group counseling sessions given concerns regarding confidentiality and data entry burden - Pilot test revised measurement-based care protocol in additional cognitive interviews	

Each column corresponds to responses from a single opioid treatment program leader, clinical supervisor, and front-line counselor, respectively. Partner feedback is collected, reviewed, and analyzed by the research team to inform strategic changes to measure content and administration procedures

The interviews have identified specific items, instructions, and response options that may require modification to enhance clarity. Specifically, partners have suggested shortening items due to confusing clinical wording to enhance literacy, rephrasing instructions using simpler language, and including a mix of open-ended and multiple-choice response options. Additionally, interviews have identified questions that can likely be removed due to limited perceived utility, conceptual overlap with other items, and fit with counseling procedures at opioid treatment programs. Perhaps most valuably, the interviews conducted thus far have elucidated partner preferences regarding ideal measure administration procedures. Specific administration advice elicited by the interviews has included: administration of the measure prior to individual or group counseling sessions, review of the measure at the start of a clinical encounter to guide service provision, and use of paper and pencil to facilitate administration off-line or in group contexts. The interviews have also provided encouraging preliminary data that the measure is viewed as low burden to be pragmatic within the standard opioid treatment program workflow. Final decisions about which items to eliminate, add, or modify, as well as how to administer the measure in the usual opioid treatment program workflow, will be made once data collection is complete to ensure responsiveness to the elucidated feedback.

## Reflections on use of cognitive interviewing

Methods to develop pragmatic measures are critical to advance implementation science [23]. As the field evolves, ensuring that partners share a common understanding of implementation constructs is essential to further the study of implementation strategies and outcomes [38]. Although cognitive interviews can be time and labor intensive, involving partners in measure development incorporates the perspectives of the end-users, which can increase measure relevancy, increase the buy-in of front-line staff and administrators, and optimize a measure’s fit within a specific organizational context. Additionally, while interviews elicit discrepant data on measure quality and fit, cognitive interviews allow researchers to qualitatively capture discrepant partner viewpoints. This increased buy-in may result in measures that are more pragmatic, easily implemented, and sustained in community-based settings.

Cognitive interviewing can facilitate a shared understanding between partners and measure developers of implementation constructs, which with time, can reduce the field’s reliance on home grown implementation measures developed for single use. We assert that using cognitive interviewing to engage partners is complementary to psychometric testing because it increases measure utility and, thus, urge implementation researchers to routinely adopt this method. We believe that cognitive interviewing has potential to improve the rigor of implementation measures and facilitate a greater common language for the field.

Measurement concerns in implementation science are among the most significant barriers to advancing the field. There is an immense need for pragmatic and psychometrically sound measures but there remains limited guidance on methods to develop these measures. We hope that the overview of the four-stage approach to cognitive interviewing provided in this manuscript, along with a case example of how these stages are actively being applied in an ongoing implementation study, can help to advance the development of pragmatic measures and address measurement issues in the field.

### Supplementary Information

**Additional file 1.** Measurement-Based Care Cognitive Interview Script. This file includes the Measurement-Based Care Cognitive Interview Script, Interview Table, and Suggested Follow-up Questions used in the MBC2OTP case example.

## Data Availability

Not applicable.
