# Supplementary material for: What do you think it means? Using cognitive interviewing to improve measurement in implementation science: description and case example
Source: Implement Sci Commun. 2024 Feb 14;5:14. doi: 10.1186/s43058-024-00549-0 (PMC10865651; doi:10.1186/s43058-024-00549-0)
Supplement: Supplementary file 1 — Additional file 1. Measurement-Based Care Cognitive Interview Script. This file includes the Measurement-Based Care Cognitive Interview Script, Interview Table, and Suggested Follow-up Questions used in the MBC2OTP case example. [file 43058_2024_549_MOESM1_ESM.docx]

**Measurement-Based Care Cognitive Interview Script**

*Note: The sample questions below are examples of possible questions to be asked of participants. Given the iterative nature of qualitative interviewing, the questions asked will evolve as data are collected and inform subsequent follow-up questions for future interviews.*

Welcome Script: Thank you so much for taking the time to meet with me today. I wanted to start off by giving you a brief overview of the goals of our discussion today. The goal of our study is to develop a measurement tool that can be used at each group therapy session to assess patient treatment progress and help guide treatment decisions. This type of approach is called measurement-based care, and it has been shown to be effective in research for enhancing the quality of counseling treatment received and for improving patient outcomes. Typically, a measure like this would be administered to all patients at the beginning of a counseling session so the information can be used to inform the session content. Many measurement-based care interventions used standard measures, but we want to tailor the measure to your treatment program and treatment approach so that it fits well with your day to day activities and patient population. Today’s interview will last about 60 minutes and I will be asking you to review and provide feedback about some questions we may ask patients receiving group counseling at your treatment program. I will audio-record our discussion so that I can give you my full attention without having to take notes. However, all your responses will be confidential and will not be shared with your program. Do you have any questions before we begin?

***BEGIN RECORDING**

Below is a list of questions that we may ask patients to respond to as part of measurement-based care in group counseling. I’d like you to start by taking a look at each of these questions and filling out or verbally discussing the questions in the table below. For each question, I would like to get your feedback about what the question means to you, whether the question is clear, whether it feels relevant to patients receiving group counseling, and whether you would recommend any wording changes, additions, or deletions of questions. When you are finished, I will ask you some additional questions.

**Measurement-Based Care Interview Table**

| **Question** | **What does this mean to you?** | **Is this question clear? Relevant?** | **Would you recommend any wording changes? Recommend keeping or deleting?** |
| --- | --- | --- | --- |
| 1. Have you used opioids, sedatives, or cocaine in the past 3, 6, or 12 months?   - 1. Yes   2. No |  |  |  |
| 1a. If yes, how often have you used them?   1. 5-6 times per week 2. 3-4 times per week 3. 2 times per week 4. 1 time per week 5. 1-3 times a month 6. Less often |  |  |  |
| 1b. Did you inject any of these?   1. Yes 2. No |  |  |  |
| 2. Have you drank more than 4 (women) or 5 (men) standard drinks on a single occasion of 2 hours or less in the past 3 months? A standard drink consists of…   - 1. Yes   2. No |  |  |  |
| 2a. If yes, how often did you drink this amount?   1. 5-6 times per week 2. 3-4 times per week 3. 2 times per week 4. 1 time per week 5. 1-3 times a month 6. Less often |  |  |  |
| 3. What typical dose of opioids do you take? |  |  |  |
| 4. Do you use any other opioids?   - 1. Yes   2. No   What opioids? |  |  |  |
| 5. What is your motivation for seeking the effect of opioids? |  |  |  |
| 6. When do you typically experience symptoms of withdrawal? |  |  |  |
| 7. Where are you typically when you experience symptoms of withdrawal? |  |  |  |
| 8. Do you take any drugs to avoid or manage feelings of withdrawal?   - 1. Yes   2. No   What drugs? |  |  |  |
| 9. Where do you typically obtain opioids? |  |  |  |
| 10. Do you use opioids with other people?   - 1. Yes   2. No   What people? |  |  |  |
| 11. What thoughts and beliefs make you want to purchase opioids? |  |  |  |
| 12. Have you ever taken any actions to avoid using opioids?   - 1. Yes   2. No   Were these actions successful?   1. Yes 2. No   Why or why not? |  |  |  |
| 13. Have you ever experienced problems as a result of the time you have spent to get opioids?   - 1. Yes   2. No   What kinds of problems? |  |  |  |
| 14. Have you ever had any negative experiences while using opioids or after?   - 1. Yes   2. No   What were they? |  |  |  |
| 15. How strong is your typical urge to use opioids?   - 1. 0 – Not at all   2. 1   3. 2   4. 3   5. 4   6. 5 – Moderate   7. 6   8. 7   9. 8   10. 9   11. 10 – Extreme |  |  |  |
| 16. What types of situations or feelings cause you to want to use opioids? |  |  |  |
| 17. Have you ever been in any physically dangerous situations while using opioids (for example like driving or operating machinery)?   - 1. Yes   2. No   What kinds of situations? |  |  |  |
| 18. Do you have any known physical health problems that are affected by your opioid use?   - 1. Yes   2. No   If so, what are they? How does your opioid use make them worse? |  |  |  |
| 19. Has your opioid use ever impacted your personal roles at home, work, or school?   - 1. Yes   2. No   If so, what impact has opioid use had on your roles? |  |  |  |
| 20. Has anyone close to you been affected by your opioid use?   1. Yes 2. No |  |  |  |
| 21 How often does your opioid use cause problems for you at home, work, or school, or with those close to you?   1. 5-6 times per week 2. 3-4 times per week 3. 2 times per week 4. 1 time per week 5. 1-3 times a month 6. Less often |  |  |  |
| 22. Have you reduced or given up any activities because of your opioid use?   - 1. Yes   2. No   If so, what activities? |  |  |  |
| 22a. Do you think there’s any way to help you restart these activities?   1. Yes 2. No |  |  |  |

[Upon finishing reviewing the table]

**For the Interviewer: *Indicates recommended language for the interviewer to read when transitioning between sets of questions. As much as possible, please read questions verbatim from the interview guide and ask all questions as time allows. Always begin with an open ended question, and then provide clarification or response options if the interviewee would like more detail/if the question is unclear to the interviewee.**

*Now that you have completed the question set, I would like to ask some follow up questions about your experience.*

**Core Concepts for Follow Up Questions**

**1. Question Content**

*My first set of follow- up questions is about the question content.*

- What are your thoughts about the measure/question content?
  - Any questions that should be added? Anything that is missing?
  - How well do you feel the questions captured the important things to ask to assess treatment progress?
  - How might responses to these questions guide your approach to treatment/treatment planning (leader/provider) or your treatment session (patient)?

**2. Measure Format**

*I’m next going to ask about the format of the measure we just reviewed.*

- What are your thoughts about the measure format and response options?
  - Should additional response options be added? If so, what options?
  - What are your thoughts on free response, multiple choice, or yes/no responses?
  - Any recommendations for changing the response options for specific questions?
- What are your thoughts on the measure length?
  - How long do you think it will take patients to complete this measure on average?
  - How long would you prefer that the measure take?
  - What (if any) questions would you remove/not ask to reduce the length/amount of time to complete the measure? *For interviewer: You can display the question table again to remind the interviewee of the questions.*

**3. Measure Administration**

*Now I’d like to ask you about your thoughts on how this measure might be administered to patients at your treatment program.*

- What are your thoughts about measure administration procedures?
  - For example, how frequently should patients complete a measure like this?
- How easy or difficult do you feel it would be to administer this measure to patients/clients in group counseling?
- What would be your recommended procedure for how information gained from this measure would be used (i.e. after it is administered) in group counseling?

**4. Measure Fit for Group Counseling**

*I want to now follow up a bit more on the use of the measure within group counseling.*

- How do you feel a measure like this would fit into a typical group counseling session at your treatment program?
  - Should this measure be completed prior to or during a group counseling session?
  - How could this measure be used in a group counseling session to inform treatment?
- How do you feel a measure like this would work for the patient/client population at your treatment program?

**5. Current treatment workflow and electronic health record procedures (leaders and providers only)**

*Finally, I’d like to ask you a bit more about workflow and electronic health record procedures at your program. I ask about workflow and health records because measurement-based care is often integrated into the electronic health record so counselors can document patient responses, and track changes to responses over time to see treatment progress.*

- What is the current treatment workflow for new and existing clients at your treatment program (e.g. process/timeline for assigning counselors, number of group/individual counseling sessions per week)?
- What electronic health/medical record does your site currently use? Has your treatment program customized any components of the electronic health record?
  - How easy or difficult do you feel it would be to make a measurement-based care tool available for use in the electronic health record (e.g. list of the questions, ability to enter responses into the record, viewing progress over time)
